# Transmission of SARS-CoV-2, Required Developments in Research and Associated Public Health Concerns

**DOI:** 10.3389/fmed.2020.00310

**Published:** 2020-06-09

**Authors:** Suliman Khan, Jianbo Liu, Mengzhou Xue

**Affiliations:** ^1^Department of Cerebrovascular Diseases, The Second Affiliated Hospital of Zhengzhou University, Zhengzhou, China; ^2^Department of Respiratory Diseases, The Second Affiliated Hospital of Zhengzhou University, Zhengzhou, China

**Keywords:** coronavirus outbreak, medical consequences, prevention, challenges, infectiousness

## Abstract

Severe acute respiratory syndrome coronavirus 2 (SARS-CoV-2) is rapidly spreading across the world to cause thousands of mortalities each day. Poor responses from the authorities to the spread of infection, lack of effective measures for prevention, unavailability of promising treatment options, and sufficient diagnostic options have created an alarming for the world. The transmission routes from human to human of SARS-CoV-2 can be the direct transmission, droplet inhalation transmission, contact transmission, transmission through saliva, and transmission via fecal–oral routes. Due to the asymptomatic spread of SARS-CoV-2's, developing control and prevention measures is challenging. Implementing proper strategies addressing the infection control and clinical supplies, understanding the mechanism associated with pathogenesis, advancing in preventive measures and effective treatment and diagnostic options are necessary to control the ongoing pandemic. In this article, we briefly discuss the features, entry mechanism, infectiousness, and health consequences related to the COVID-19 outbreak.

## Introduction

SARS-CoV-2 has infected over five million people worldwide after its emergence in Wuhan, China (1). The world has witnessed that this virus can spread rapidly to cause the death-causing COVID-19 disease. Although the rate of recovery is higher in people with strong immune responses, however, the immune-compromised individuals are at higher risks to be readily killed by the infection (2). The major reasons for higher morbidity and mortality rates are rapid human-human transmission, unavailability of promising diagnostic and therapeutic options, scarcity of clinical supplies, shortage of medical and clinical staff, and lack of effective preventive measures (3). Besides the physical illness, the COVID-19 epidemic has also increased the risk of psychological problems among healthcare workers, infected individuals, and the general public (3, 4), due to the fear of treatment failure, higher morbidity and mortality, lack of psychological interventions, and infodemia (3, 5, 6).

During the early days of the epidemic in China, a number of countries suspended travel to and from China, evacuated their nationals from the epicenter, and placed them in quarantine to curb the risks of pandemic (6). These responses were not sufficient to prevent the spread of COVID-19, therefore, it became a global pandemic (7). Considering the seriousness of this situation scientists and medical researchers came forward and extended their services to the development of therapeutic strategies, preventive measures, and strategies to control the unfolding pandemic. Until now, researchers have unveiled some of the important biological and clinical features for COVID-19 infection, including the characterization of the whole genome (8) and spike glycoproteins (9), investigation of clinical features and evaluation of different broad-spectrum antiviral drugs in combination with either antibacterial, antimalarial and/or traditional Chinese medicines (10). Nevertheless, more research work is required to further investigate the sources of transmission, the biology of viral incubation and reemergence, and the potential of vertical transmission from mothers to neonates. In this article, we discuss the features of coronaviruses, the mechanism of infectiousness of SARS-CoV-2, and its medical consequences. We also describe the populations at higher risk and challenges in research progress. This narrative review article will benefit the public and scientific community regarding the current progress and the need for further work.

## Methodology

To identify and select the papers in this review we searched the published research and review articles relevant to origin and outbreaks of three human coronaviruses, and features, transmission, spread, entry mechanisms, infectiousness, control strategies, and animals hosts for SARS-CoV-2. We also search the papers published on SARS and MERS coronaviruses in the aspects of animal models and sources of transmission. We reviewed the World Health Organization, U.S. Centers for Disease Control and Prevention, Nature reports, Medline, PubMed Central, Embase, google scholar, and ScienceDirect, according to the relevancy as explained earlier, until April 20, 2020. The search terms “novel coronavirus, SARS-CoV-2 and COVID-19, SARS and MERS” were broadly used. Studies conducted in laboratory and clinical based observations, and/or conducted through bioinformatics techniques were included.

## Clinical Features of COVID-19

Pneumonia is one of the most frequent manifestations of COVID-19 infection, which is characterized by fever, bilateral infiltrates on chest imaging, cough, and dyspnea (11). The period from infection to symptoms appearance ranges from 2 to 14 days, while the average period reported so far is ~5 days (12). One of the previous studies reported the onset of fever and respiratory symptoms ~3–6 days in a family cluster of infections (13). Similarly, in an analysis of 10 patients with confirmed COVID-19 pneumonia, the estimated mean incubation period was 5 days (11). Furthermore, the majority of the individuals showed moderate symptoms whereas 20% of the infected patients showed severe illness of respiratory failure and septic shock and gastrointestinal complications (11, 13). Common laboratory abnormalities associated with COVID-19 are lymphopenia and elevated aminotransferase levels (10). C-reactive protein (CRP) levels have been reported to alter with the development of symptoms, such that patients with severe pneumonia present high CRP levels (10, 14). In a recent study, Wang (14) reported that CRP levels at the early stage of COVID-19 are positively correlated with lung lesions and symptoms development, which can be used as one of the key indicators for disease development and severity. Wang et al. (10) investigated 138 patients [median age; 56 years, interquartile range; 42–68 years] with COVID-19 pneumonia in Wuhan and reported that 136 patients developed fever, 82 patients had a dry cough and 96 patients had fatigue. Besides lymphopenia, parenchymal lung abnormalities were also common among all patients as depicted from computed tomography of the chest, including bilateral patchy shadows or ground-glass opacities. Nonetheless, some people have been reported to be initially asymptomatic and may remain asymptomatic or go on to develop disease on later stages (WHO; March 23, 2020). Although it is important to know about the symptoms' appearance and severity, however, understanding the transmission of the infection to healthy individuals from COVID-19 patients and zoonotic sources can be of great importance in the aspects of developing strategies to prevent and control the spread of COVID-19.

## Emergence and Transmission of Coronaviruses

During November 2002, a novel coronavirus caused SARS epidemic in Guangdong, China (15), followed by subsequent outbreaks in Hong Kong (15, 16). This outbreak was reported to be caused by SARS-CoV, originated from market civets before its transmission and infection in humans (17). By the end of the epidemic, SARS-CoV infected 8,098 people and caused 774 fatalities in 29 different countries (16). Later on, during June 2012 a patient infected with MERS-CoV developed severe pneumonia and died in Jeddah, Saudi Arabia (16, 18), following by series of clustered outbreak in the Middle East and several other countries (16, 19). Before transmitting into humans, MERS-CoV originated and replicated in dromedary camels (17). Until 2020, MERS-CoV infected 2,468 individuals and caused 851 fatalities worldwide (20, 21).

In December 2019, clusters of patients reported with COVID-19 caused by SARS-CoV-2 were epidemiologically found linked to animals and the seafood selling market in Wuhan, China (22). The zoonotic source of its origin and transmission is still debatable, however, some reports suggested bats (23) as the possible sources of transmission (9). The human-to-human transmission of SARS-CoV-2 is thought to occur mainly via respiratory droplets produced by coughing or sneezing from an infected individual (24). The rapid increase in suspected as well-confirmed cases has also been inferred with viral transmission through the fecal-oral route and aerosol formation. The half-life on the surfaces of stainless steel, copper, and cardboard is ~5.8 h, while that on the plastic surface is 6.8 h (25). Moreover, several reports have confirmed the asymptomatic transmission while there is a chance for the animal to humans transmission (26). Overall, these observations indicate that appropriate care is necessary while handling both confirmed and suspected individuals. Moreover, the surfaces of potentially virus-contaminated places, objects, and containers should be cleaned with effective disinfectants.

## Infectiousness and Cellular Entry of SARS-CoV-2

The SARS-CoV-2 contains a single-stranded RNA with 29,891 nucleotides, encoding for 9,860 amino acids (27). The spike glycoproteins of SARS-CoV-2 contain two subunits (S1 and S2) (8). The S2 subunit contains transmembrane and cytoplasmic domains along with fusion peptide. Novel coronavirus has over 80% identity with SARS-CoV. However, spike receptor-binding domains (RBD) are only 40% identical (28), while structural elements open reading frame (ORF)3b and ORF8 were found with no homology (29). Coronaviruses contain six ORFs regions which serve as templates for the production of sub-genomic mRNAs and encode protein, spike, nucleocapsid, and membrane proteins. ORFs are responsible for the production of pp1a and pp1ab polypeptides (30). Both SARS-CoV and MERS-CoV infect bronchial epithelial cells and type II pneumocytes through ACE2 and CD26 receptors, respectively (17, 31, 32). The mechanism associated with the infectiousness of SARS-CoV-2 is yet to investigate, however, it likely infects the bronchial cells through ACE2.

**Figure 1:**
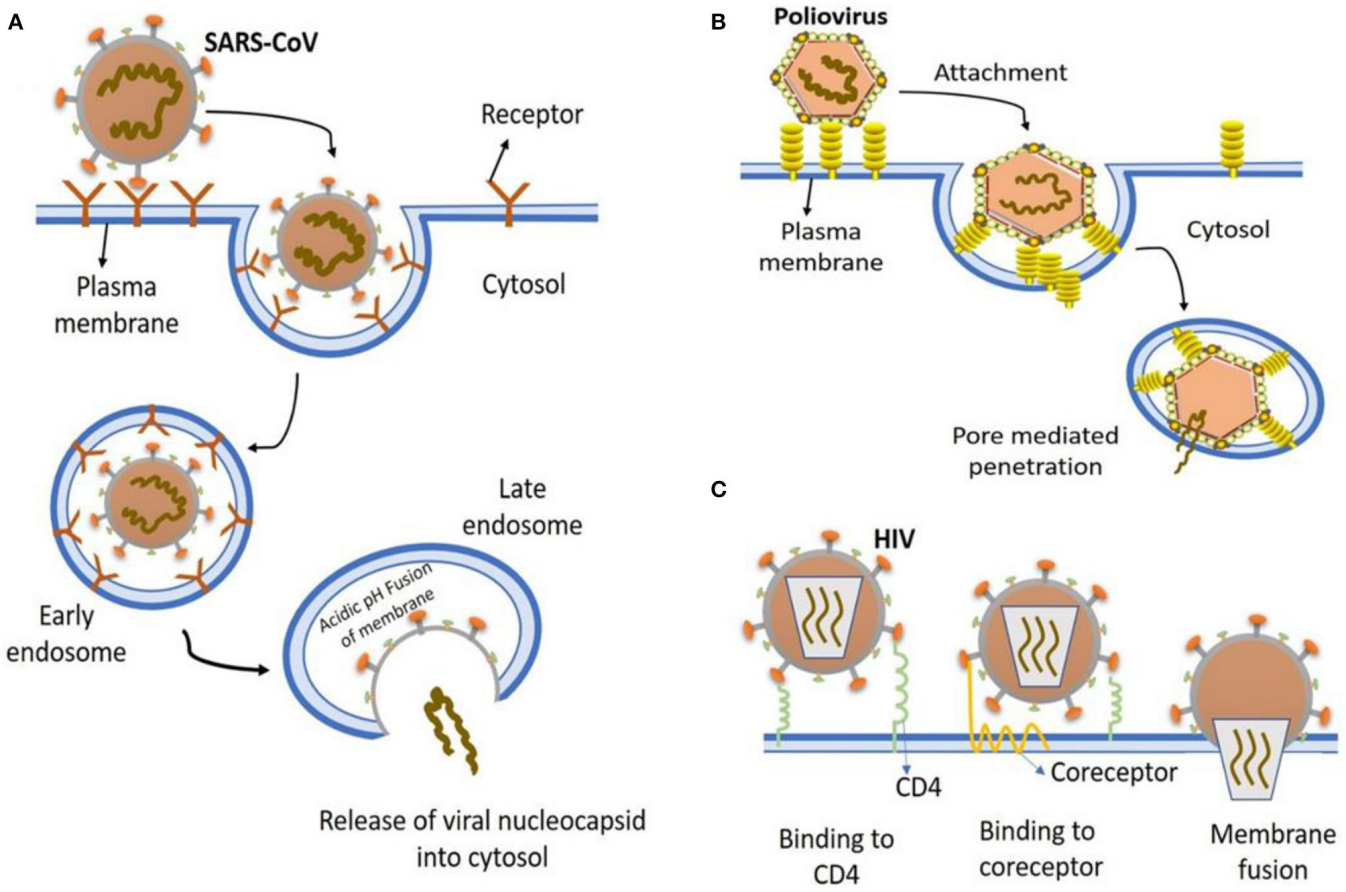
The most common entry to host cells mechanisms of human viruses. **(A)** SARS-CoV entry. Key points are, virion attachment to receptor; virion internalization by endocytosis; lowering the pH (5.5) of the endocytic vacuole leading to drastic reconfiguration of the viral attachment protein; insertion into the vacuolar membrane; fusion of vacuolar membranes and the viral; viral nucleocapsid release into the cytosol. **(B)** Poliovirus entry. virion binding to cell surface receptors, endocytosed and ultimately delivered to endosomes (low pH); conformational changes in viral capsid due to low pH environment result in exposure of hydrophobic domains that insert into the endosomal membrane, producing a pore for viral genome exit and entry into cytoplasm. **(C)** HIV entry. Virion attaches to various attachment factor on cell surface, such as DC-SIGN. The attachment of viral envelope glycoprotein to CD4 alters the structure of envelope glycoprotein, which then induces the second receptor binding domain exposure resulting in the engagement of CCR5 or CXCR4 coreceptors, that in turn causes the viral fusion with the cell membrane.

**Figure 2:**
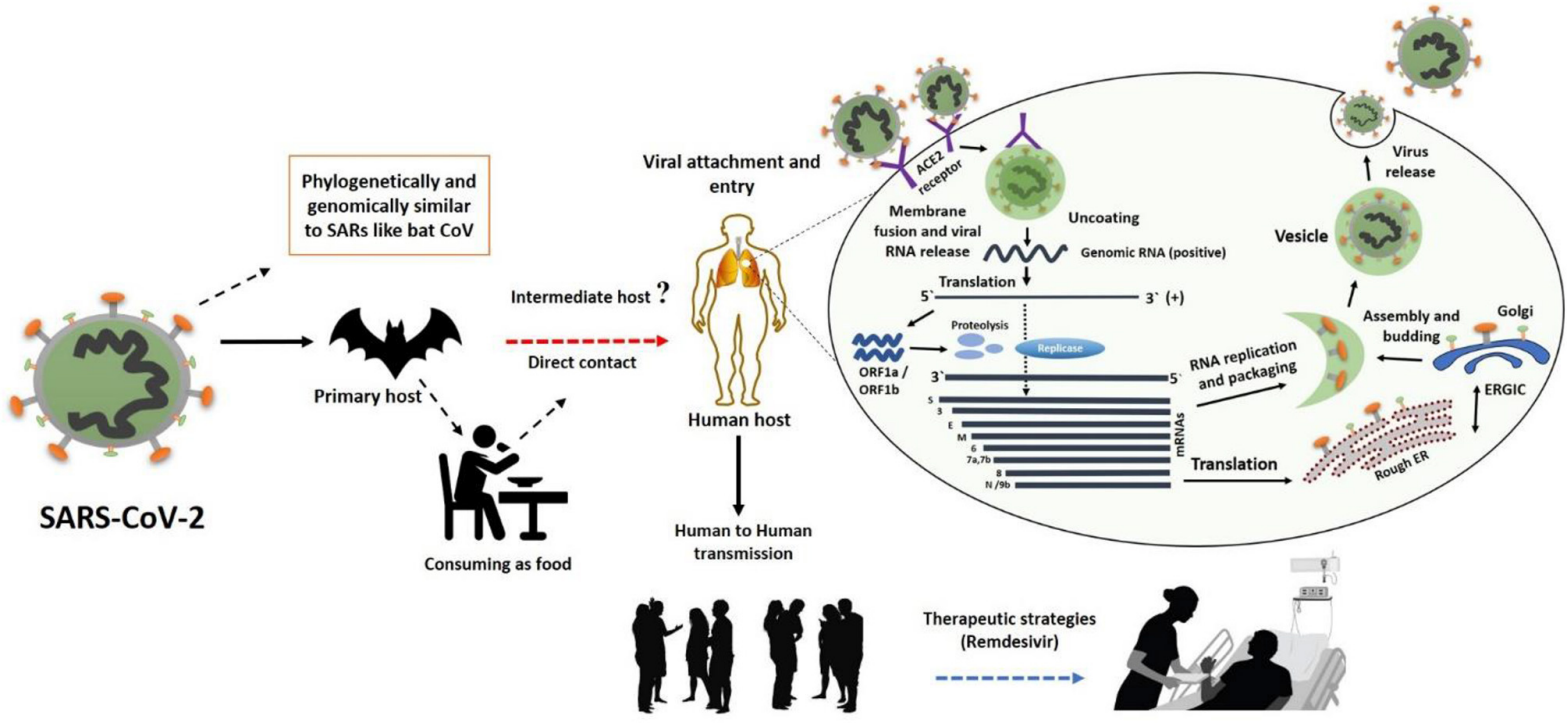
The SARS-CoV-2 transmission from bats via unknown intermediate to humans causes infectiousness known as COVID-19 disease. The binding of S protein to ACE2 receptor initiates the life cycle which is then followed by conformational changes in the S protein, which further facilitates the fusion of viral envelope and host cell membrane. Following the fusion through endosomal pathway, SARS-CoV-2 then releases RNA into the host cell, which is translated into pp1a and pp1ab. Next, viral proteinases cleave the translated proteins into small products, meanwhile a series of sub-genomic mRNAs are produced by polymerase enzyme through discontinuous transcription, which are then translated into specific viral proteins. These viral proteins and genome RNA are assembled to form virions in Golgi and endoplasmic reticulum, which are later transported out of the cell via vesicles. This figure was designed by updating and modifying the information from our previously published paper (29).

In general, a virus entry to the host cell comprises a series of fundamental interactions; (i) binding to a target host cell via cellular receptors; (ii) fusing the envelope with a cellular membrane; and (iii) forking over its genetic material inside the cell (Figure 1). The process of viral genomic delivery of nucleic acids into the host cell is highly dependent upon binding specificity to receptors, proteolytic activation, and endocytosis efficiency (33, 34). Coronaviruses demonstrate a great degree of plasticity regarding the entry pathways, which can occur at the plasma membrane or through the endocytic pathway (35) (Figure 2). The entry to the host cell process of SARS-CoV-2 is regulated by Glycosylated spike (S) fusion protein and host receptor known as ACE2. The S proteins is capable of significant structural rearrangement thus, play a crucial role in fusing the viral membrane with the host cell membrane (36). This fusion process sparks off with binding of the S1 subunit to ACE2 and is linked with the accessibility of receptor determined by hinge-like conformational movements of the receptor-binding domain (RBD) of S1. Thus, RBD can transiently hide or expose the determinants of receptor binding through receptor-inaccessible state or receptor-accessible state, respectively (37). Once the virus has entered to the host cell, the replication-transcription complex (RTC) is organized in double-membrane vesicles to initiate transcription of polyprotein 1a/1ab (pp1a/pp1ab). These pp1a/pp1ab proteins encode chymotrypsin-like protease (3CLpro), main protease (Mpro), and papain-like proteases for the production of non-structural proteins (nsps) (28). Trans-membrane helical segments in the ORF1ab region encodes for nsp2 and nsp3 (38). The structural proteins and nsps play a role in the pathogenicity of SARS-CoV-2 by blocking the innate immune response and assembly and release of newly synthesized virions (39).

During the first days of the Wuhan epidemic, two strains of novel coronavirus were reported namely S strain and L strain. Observations suggested that L strain was more aggressive and more fatal as compared to S strain. A group of researchers from Pasteur Institute Shanghai and Peking university reported that the rate of infection for L strain was as high as 70%, while that of S was ~30% as indicated by the analyzed samples. On the other hand, S type strain was found to be the ancestral version and was closely related to viruses like TG13. Further analysis based on population genetics indicated that these strains mainly differed at orf1ab and ORF8 regions. Interestingly, the development of new variations of the spike protein in SARS-CoV-2 variants is linked to mutations, and natural selection (40). Therefore, further studies should evaluate the combinational impacts of genomic data, epidemiological data, and chart records of the clinical symptoms of patients with COVID-19.

## Current Research Gaps Associated With COVID-19 Transmission

After the identification of SARS-CoV-2, debates started among scientists on its sources of origination and zoonotic source of transmission to humans (29). The identity of the animal source of SARS-CoV-2, is still one of the key missing gaps that scientists are being racing to investigate. It is a known fact that coronaviruses circulate in mammals and birds (17), and researchers have already suggested bats to be the source of origination for SARS-CoV-2 (23). However, an intermediate animal was probably the source of transmission of the virus to humans. Early claims came from researchers related to intermediate sources of transmission faced controversies (9). A recent report discredited an earlier statement that pangolin could be the possible intermediate source that might have received the virus from the bat and transferred it to humans (40). According to more recent study on molecular and phylogenetic analyses, it is unlikely that SARS-CoV-2 emerged directly from the pangolin coronaviruses (41), suggesting that pangolins may not be responsible for the transmission of SARS-CoV-2 to humans.

With further spread of the virus after its outbreak in Wuhan, more people became infected, thus, human to human transmission became more evident. One of the reasons for the high rate of infectiousness in humans is thought to be the higher affinity of RBD for binding to ACE2 receptors (29, 42). In addition, the determination of host range and binding to the ACE2 are highly dependent on six RBD amino acids “L455, F486, Q493, S494, N501, and Y505 in SARS-CoV-2” in SARS-CoV-2, thus, RBD can also bind to ACE2 from ferrets and cats (42). On the other hand, the high-affinity of RBD to human ACE2 is thought to be linked with natural selection on a human ACE2, indicating that SARS-CoV-2 was not produced with purposeful manipulation (42). These observations support the hypothesis that SARS-CoV-2 was transmitted from a yet unknown intermediate zoonotic source to humans.

Spike glycoproteins have been well-documented in the aspects of transmission and entry of SARS-CoV-2 into host cells (13, 28). It is notable that the polybasic cleavage site of SARS-CoV-2 at the junction of S1 and S2 allows cleavage by proteases such as furin, which plays a crucial role in infectiousness and determining host range. Despite the unknown functional consequence, the higher genetic variation in spike indicates that SARS-CoV-2 with polybasic cleavage sites may be discovered in several other species (42, 43), which can be the possible source of transmission for SARS-CoV-2 to humans. Interestingly, the mutation found in the polybasic cleavage site was not related to that of the bat and pangolin viruses (42), therefore, it may be linked with the virus's ability for transmission and infection in humans. The determination of polybasic cleavage and predicted O-linked glycans further suggest that the virus was most likely transferred from an animal with ACE2 to humans, as these are not possible in cell cultures (42). Further research to determine the impact of polybasic cleavage and predicted O-linked glycans on transmissibility and pathogenesis is necessary.

Although investigating the mechanisms underlying entry to host cell, transmission, polybasic cleavage, and predicted O-linked glycans are required to determine the research gaps associated with transmission and origination, however, this work requires suitable animal models. Unfortunately, there is no promising model while the non-human primates tested for SARS and MERS were unable to develop severe diseases in response to the infectiousness (44). Nevertheless, the models developed for the expression of human ACE2 and DPP4 (16) can be further modified and used to study the transmission and infectiousness of SARS-CoV02. Moreover, CRISPR-interceded genetically modified small animals can be also utilized for the study of the pathogenicity of SARS-CoV-2. Nevertheless, it is important to investigate the ultimate source of viral transfer to humans, as even if the virus is eradicated with social distancing, other sources including zoonotic and environmental sources can again cause the transfer into humans, and thus another outbreak will be the result.

## Major Health Concerns Associated With COVID-19

The ability of rapid human to human transmission of COVID-19 infection especially through asymptomatic infected individuals and aerosol, has paralyzed life across the globe (29, 43). Although the COVID-19 infection primarily affects physical health, however, it can also affect mental health through the fear of transmission from unknown sources and high mortality rate that can further paralyze life (5). It is deemed necessary that timely effective services should be provided to the vulnerable populations as reported by Khan et al. (5). The adverse impacts of COVID-19 are specific to the populations, therefore, we discuss the most vulnerable populations, the current evidence on known vulnerable groups and the associated health risks in response to the COVID-19 infection.

Rapidly increasing mortalities and morbidities in healthcare workers are causing serious medical concerns and adversely affecting healthcare services worldwide (3). The fear of being infected due to close contacts with infected symptomatic and asymptomatic patients, and prolonged working schedules may decrease the working efficiency in current doctors and nurses (3, 7). A large number of medical and clinical staff are likely to be infected with COVID-19 infection. Only in Wuhan, more than 15 hundred persons from healthcare settings were reported infected (3). In addition to the high risk of contracting infection due to direct interaction with infected and suspected individuals (3), healthcare workers have also been reported to develop severe mental conditions including stress, anxiety, and related mental illnesses (3, 4). To mitigate the risk of contracting infection the medical staff should adhere to standard precautions while providing patient care (45, 46).

According to the CDC report on coronavirus disease, individuals with underlying chronic medical conditions are at higher risk for contracting COVID-19 infection. Huang et al. reported that 32% of SARS-CoV-2 infected individuals had diabetes, hypertension, and cardiovascular disease (11). The fatality rate was also high in individuals who had diabetes mellitus, chronic lung disease (47), cerebrovascular diseases (48), and hypertension (47). Furthermore, COVID-19 infection in patients with lung cancer can develop severe COVID-19 disease that can lead to death (49). Luo et al. (49) reported that more than half of the COVID-19 infected individuals who had lung cancer, needed hospitalization, whereas nearly a quarter of them died. However, people living with human immunodeficiency virus do not present excess morbidity and mortality among symptomatic COVID-19 patients (50). The higher risk of disease and death in individuals with underlying diseases might be linked with weaker or comprised immune responses.

The elder individuals are comparatively more affected by COVID-19 infection; however, individuals of any age can acquire the infection (51). According to the previous reports, 87% of infected individuals were between 30 and 79 years old. Moreover, the mortality rate was higher in older people. The case fatality rate of 8% was observed among individuals having age between 70 and 79 years, while 15% fatality rate was reported in people with 80 years or older (47).

COVID-19 infection in pregnant women is of serious concern, as it might have detrimental effects not only on mother's health but also on neonatal health can be at risk (52). In a recent study, COVID-19 infection was found to cause adverse neonatal outcomes. Two of the neonates were tested positive, and for COVID-19 while, five were found with neonatal pneumonia, suggesting the possibility of a link between adverse pregnancy outcomes and COVID-19 infection (52). Dong et al. (53) reported a newborn with elevated IgM antibodies to SARS-CoV-2, who was born to a mother with COVID-19, suggesting the possibility of vertical transmission. Therefore, further investigations should focus on adverse pregnancy outcomes and the possibility of vertical transmission. The approach to prevention, evaluation, diagnosis, and treatment of pregnant women with suspected COVID-19 should be similar to that in non-pregnant individuals, with the consideration that pregnant women with other potentially severe respiratory infections, such as influenzaappear to be more vulnerable to developing severe sequelae. Moreover, pregnant women should be given attention and provided with the utmost facilities in terms of treatment and diagnosis.

## Controlling The Spread of COVID-19

Controlling the spread and transmission of infection is one of the major issues that authorities are currently considering with serious attention. World Health Organization (WHO) and U.S. Centers for Disease Control and Prevention (CDC) recommend face and eye protection for droplet and contact precautions. During aerosol-generating procedures, such as non-invasive ventilation, tracheotomy, cardiopulmonary resuscitation, tracheal intubation, bronchoscopy, and manual ventilation before intubation, additional precautions are warranted such as airborne infection isolation room and wearing the appropriate personal protective equipment (ref 1 and ref 2).

To control the transmission requires the identification and isolation of the infected individuals. Samples from the nasopharyngeal swab, oropharyngeal swab, sputum, tracheal aspirate, or bronchoalveolar lavage should be tested for the detection of the virus (54). The symptoms of COVID-19 pneumonia are primarily similar to influenza and seasonal allergies (10, 55, 56), therefore using thermo-scanners and physical observations are not are not able to adequately differentiate between those conditions. Although quantitative real time polymerase chain reaction (qRT-PCR) is the major confirmatory test however, to provide further testing support developing additional testing kits that could rapidly detect SARS-CoV-2 with maximum accuracy in suspected, confirmed, and asymptomatic patients may be useful.

To control the ongoing pandemic and risk of future epidemics, the development of safe and effective vaccines is necessary, that should be available for individuals at high risk of contracting COVID-19 infection. Until now, effective vaccine against COVID-19 is not available, however, some vaccines with preventive potential against COVID-19 infection are in pipeline. Such as the mRNA-based vaccine developed by National Institute of Allergy and Infectious Diseases in USA, is being trialed (57). While the INO-4800-DNA based vaccine is currently being developed (57). Moreover, Center for Disease Control and Prevention (CCDC) in China has started working on inactivated virus vaccine that may be used widely if found promising (57, 58). Stermirna Therapeutics has reported the development of mRNA-based vaccines that can soon be available for trials (57, 58). Nevertheless, more work is required; SARS-CoV specific live-attenuated (16) and rhesus θ-defensin 1 and protein cage nanoparticles based vaccines can be evaluated for COVID-19 infection (59, 60). Moreover, monoclonal antibodies should be considered that are effective in inhibiting virus-cell receptor binding and virus-cell fusion (16).

## Conclusions and Perspectives

SARS-CoV-2 is likely originated in bats and introduced to the world through a yet unknown intermediate. Without finding the missing intermediate, SARS-CoV-2 may reemerge even if the current spread is controlled completely through social distancing and isolation. An earlier report that indicated pangolins as the possible source of transmission of SARS-COV-2 has been discredited, therefore, further work is required to identify the unknown intermediate animal source that caused the transmission of the virus to humans. Based on their role in transmission and infectiousness, spike glycoproteins, RBD binding to ACE2 and mutations in polybasic cleavage sites related to different animals should be studied further.

Given the importance of the current outbreak in Wuhan, further studies are necessary to provide deep understating of replication, pathogenesis, and biological properties using the relevant biological techniques such as reverse genetics and molecular techniques. To unveil pathogenesis and entry mechanisms further investigations should focus on structural elements ORF3b and ORF8 in novel coronavirus. These regions may play an important role in high human to human spread and may be linked to the severity of the disease. These investigations will help the control and prevention of COVID-19 mediated pneumonia and novel emerging diseases in the future. The COVID-19 outbreak has affected millions of people around the globe by causing mortalities and morbidities. Thus, curbing COVID-19 and preventing it from spreading further requires the development of effective strategies t related to detection of the virus, curing the disease, vaccination and prevention, and identification of the transmission sources. The research work should focus on preventing the spread and transmission of the virus, however, without taking effective measures the virus will come back again.

## Author Contributions

SK is the leading author while JL and MX contributed to revisions. All authors contributed to the article and approved the submitted version.

## Conflict of Interest

The authors declare that the research was conducted in the absence of any commercial or financial relationships that could be construed as a potential conflict of interest.
